# Pilot study: Application of artificial intelligence for detecting left atrial enlargement on canine thoracic radiographs

**DOI:** 10.1111/vru.12901

**Published:** 2020-08-11

**Authors:** Shen Li, Zigui Wang, Lance C. Visser, Erik R. Wisner, Hao Cheng

**Affiliations:** ^1^ William R. Pritchard Veterinary Medical Teaching Hospital, School of Veterinary Medicine University of California Davis California USA; ^2^ Department of Animal Sciences University of California Davis California USA; ^3^ Department of Medicine and Epidemiology, School of Veterinary Medicine University of California Davis California USA; ^4^ Department of Surgical and Radiological Sciences, School of Veterinary Medicine University of California Davis California USA

**Keywords:** artificial intelligence, convolutional neural networks, myxomatous mitral valve disease

## Abstract

Although deep learning has been explored extensively for computer‐aided medical imaging diagnosis in human medicine, very little has been done in veterinary medicine. The goal of this retrospective, pilot project was to apply the deep learning artificial intelligence technique using thoracic radiographs for detection of canine left atrial enlargement and compare results with those of veterinary radiologist interpretations. Seven hundred ninety‐two right lateral radiographs from canine patients with thoracic radiographs and contemporaneous echocardiograms were used to train, validate, and test a convolutional neural network algorithm. The accuracy, sensitivity, and specificity for determination of left atrial enlargement were then compared with those of board‐certified veterinary radiologists as recorded on radiology reports. The accuracy, sensitivity, and specificity were 82.71%, 68.42%, and 87.09%, respectively, using an accuracy driven variant of the convolutional neural network algorithm and 79.01%, 73.68%, and 80.64%, respectively, using a sensitivity driven variant. By comparison, accuracy, sensitivity, and specificity achieved by board‐certified veterinary radiologists was 82.71%, 68.42%, and 87.09%, respectively. Although overall accuracy of the accuracy driven convolutional neural network algorithm and veterinary radiologists was identical, concordance between the two approaches was 85.19%. This study documents proof‐of‐concept for application of deep learning techniques for computer‐aided diagnosis in veterinary medicine.

## 1 INTRODUCTION

Approximately 10% of canine patients presented to general veterinary practitioners have heart disease.^1^ Myxomatous mitral valve disease, the most common acquired progressive cardiac disorder, accounts for approximately 75% of these patients.^1^, ^2^ Dogs with myxomatous mitral valve disease initially develop degenerative lesions of the mitral valve that can lead to left atrial enlargement and congestive heart failure in some dogs. Boswood et al showed that administration of Pimobendan to patients with echocardiographic left atrium and left ventricle enlargement but not yet in heart failure delays the onsite of congestive heart failure for approximately 15 months, >10% of the average lifespan of most canine patients.^3^ Accurate diagnosis of myxomatous mitral valve disease, and specifically detection of left atrial enlargement, an early feature of some dogs with myxomatous mitral valve disease, is therefore essential for appropriate initial medical management and to assess risk of heart failure and prognosis.^3^, ^4^, ^5^, ^6^, ^7^

A presumptive diagnosis of myxomatous mitral valve disease is often reached based on signalment, a left apical systolic murmur, and characteristic thoracic radiographic features. It is confirmed with an echocardiographic examination. Echocardiography is relatively expensive, requires specialized training to perform accurately, and is of limited access in general practice. Currently, the clinically applicable methods to identify left atrial enlargement from thoracic radiographs include detection of characteristic cardiac margin changes, carinal elevation, subjective mainstem bronchial widening, bifurcation angle measurements, and vertebral heart score estimations.^8^, ^9^, ^10^ None of these, however, are considered consistently accurate, and radiographic interpretation of left atrial enlargement has been shown to be inconsistent, particularly when performed by those without advanced training.^11^

Thoracic radiographic examination is readily accessible and easily performed, but its value as a test for assessing left atrial enlargement varies depending on the interpretive skill of the clinician evaluating the study and the accuracy of thoracic radiographs for assessing left atrial enlargement. The use of automated diagnosis from radiographic images using deep learning, an artificial intelligence technique that may match or exceed human expert performance in recognition of highly heterogeneous diagnostic images, has recently gained traction.^12^, ^13^ Indeed, the number of peer‐reviewed articles published annually relating to convolutional neural networks or deep learning have increased exponentially over the past 5 years.^14^, ^41^ Image analysis using deep learning techniques has been used in human medicine with success to detect and stage diabetic retinopathy and to accurately differentiate radiographs of patients with tuberculosis from normal controls given a sufficiently large training dataset.^12^, ^15^ The specific deep learning approach best suited for analyzing diagnostic images is the application of convolutional neural network.^16^

Convolutional neural network is essentially a complex computer algorithm that is commonly used for image analysis. The algorithm contains multiple processing layers and many parameters; however, it is not explicitly programmed or pre‐defined, thus referred as “black box.” The parameters within the algorithm were tuned to achieve a good fit between the input (images) and classification labels (in our study, left atrial enlargement). Input data go through multiple processing layers of abstraction to match the label. When the algorithm is exposed to large amount of data, mathematically, it has higher chance to give more accurate results.

Based on our review of the literature, a convolutional neural network model has not been explored in veterinary medicine as a means of assisting imaging diagnosis but has the potential to be an affordable, rapid, and reliable tool for veterinary medical diagnosis. The purpose of this investigation was to create an automated imaging diagnostic tool using deep learning techniques and test its accuracy to that of veterinary radiologists.

## 2 MATERIALS AND METHODS

### 2.1 Dataset

Medical records from the University of California, Davis, Veterinary Medical Teaching Hospital (VMTH) from 2010 to 2017 were screened for canine patients who had a thoracic radiographic examination and a contemporaneous echocardiographic examination performed within 72 hours of the radiographic study. All radiographs were acquired using the same radiographic units with DICOM output Digital radiography system (Sound Technologies, Carlsbad, CA) and reviewed using a DICOM viewer (eFilm/Merge Healthcare, Chicago, IL). From this dataset, patients were included in the investigation if the radiographic examination included a right lateral view and the radiographic and echocardiographic examinations included a formal report reviewed by a board‐certified veterinary radiologist (American College of Veterinary Radiology) or veterinary cardiologist (American College of Veterinary Internal Medicine), respectively. Patients were excluded if the echocardiographic report was equivocal or ambiguous in determination of left atrial enlargement or if an abridged echocardiographic examination was performed with no mention of left atrial size. For assessment of left atrial size, our standard institutional echocardiographic examination imaging protocol includes a combination of subjective assessment and two linear left atrial measurements indexed to the aorta. One measurement involves the left atrium to aortic root ratio from a right parasternal short‐axis view in early‐diastole.^42^ The other left atrial size measurement involves maximum (end‐systolic) left atrial dimension from a right a standard right parasternal long‐axis four‐chamber view, which is indexed to the aortic diameter measured in systole (annulus of the maximally opened aortic valve cusps) from a standard right parasternal long‐axis left ventricular outflow tract view.^43^

Images were designated as being echocardiographically “positive” or “negative” for left atrial enlargement based on conclusions in corresponding echocardiographic reports. Similarly, images were designated radiographically positive or negative based on the corresponding radiology reports.

### 2.2 Methods

This was a retrospective pilot study. Right lateral thoracic radiographic DICOM images were downloaded as .jpeg files directly from the hospital PACS server with no initial alteration of native matrix size. Image file size ranged from 63 to 439 KB and image matrix size ranged from 1096  × 576 to 2688 × 2208 pixels depending on the size and detector density of the radiographic detector plate used during image acquisition. All annotations were removed from the native DICOM images prior to transfer. For analysis, all radiographic images were then resized to a matrix of 64 × 64 pixels consistent with matrix downsizing used in previous deep learning network publications.^12^, ^13^, ^17^ The preprocessing time, including loading and resizing, required <1 min per image. All radiographic images were assigned to either a training dataset, or a testing dataset. Images assigned to the testing dataset were used exclusively for the purpose of testing the deep learning model and were not used for training. The first 711 images (90% of total), in chronological order based on the date radiographic images were acquired, were designated as the training dataset. The remaining 81 images (10% of total) were used for testing the resulting model. Training images were randomly split into 10 training subsets of approximately equal size to train the deep learning algorithm. The training of the deep learning algorithm involved using the multiple training subsets to tune different parameters and using validation datasets to pre‐test/retune the algorithm. To better use the information from the training dataset, a strategy called cross‐validation was applied, where each training subset was sequentially used as a validating dataset with the remaining nine training subsets used for training.^18^, ^19^ The 10 well‐tuned deep learning models obtained from cross‐validation were used to evaluate the prediction performance of the remaining 81 testing dataset images with the prediction results averaged from these 10 models. The prediction results were presented as a probability, a number between 0 and 1, indicating the likelihood that a given image was positive for left atrial enlargement. When the *P* was >.5, the result was interpreted as a “positive prediction.” The results of the testing dataset were used to assess the diagnostic accuracy, sensitivity, and specificity.

A modified classical convolutional neural network model, the Visual Geometry Group 16 network, was applied in our analysis using the deep learning package Keras.^20^ Keras (version 2.3.0) is an open‐source package written in Python. The model structure for the Visual Geometry Group network is composed of 13 convolution layers, five pooling layers, one drop out layer, and two dense layers with a total of 7 861 032 parameters. The following model parameters were used for training: 32 batch size, 100 epoches, and 0.0001 learning rate. The "same padding” technique was used in the model to improve the use of pixels on the edge of the image. The Adam optimizer and 0.01 kernel regularizer were used.

Two training goals are used in our analysis. The first one is to train a model that values positive image prediction accuracy and negative image prediction accuracy equally, that is, achieving the highest overall accuracy. The second training goal is to train a model that values more on positive image prediction accuracy than negative image prediction accuracy, that is, training a model that focus more on positive image detection. Corresponding to these two goals, two distinct loss functions were used to train the model including an accuracy driven function and a sensitivity driven function. Results from our models were compared with those from veterinary radiologist reports. The congruency of the prediction results between our models and veterinary radiologists were also illustrated.

Accuracy was calculated as (A + D)/(A + B + C + D), sensitivity was calculated as A/(A + B), and specificity was calculated as D/(C + D) (Table 1).

**TABLE 1:** Contingency table for calculating sensitivity and specificity

	Predict positive	Predict negative
True Positive	A‐true positive	B‐false negative
True Negative	C‐false positive	D‐true negative

In addition, we cropped the heart regions from all original images using the following criteria: visible cranial margin of the heart, visible caudal margin of the heart, ventral margin of the spine, and dorsal margin of the sternum. We performed our analysis using the same techniques and models. The results are very similar to the one using the entire image size. The detailed results revealed that there is almost no difference between two approaches using uncropped and cropped data.

## 3 RESULTS

**FIGURE 1:**
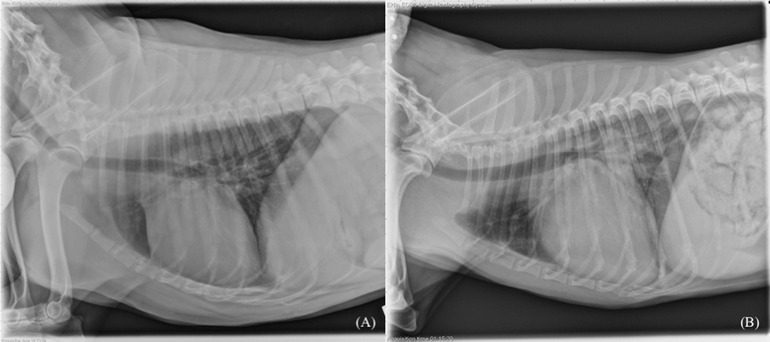
Thoracic right lateral radiographs annotated to be negative (A) and positive (B) for left atrial enlargement

Seven hundred ninety‐two patients met the inclusion criteria and were enrolled in the investigation with 281 of these echocardiographically positive and 511 echocardiographically negative for left atrial enlargement. Of these, 711 images were included in the training set (262 echo positive, 449 echo negative) and 81 were included in the testing data set (19 echo positive, 62 echo negative). Two representative images exemplifying positive and negative classifications are shown in Figure 1.

In the accuracy driven convolutional neural network model, of the 81 images in the testing dataset, 13 positive images were predicted positive, 6 positive images were predicted negative, 8 negative images were predicted positive, and 54 negative images were predicted negative using the accuracy driven convolutional neural network model (Table 2). The overall accuracy of the accuracy driven convolutional neural network model was 82.71% with a sensitivity of 68.42% and specificity of 87.09% (Table 6).

**TABLE 2:** Prediction result of the accuracy driven model

	Predict positive	Predict negative	Total
True Positive	13	6	19
True Negative	8	54	62
Total	21	60	81

In the sensitivity driven convolutional neural network model, of the 81 images in the testing dataset, 14 positive images were predicted positive, 5 positive images were predicted negative, 12 negative images were predicted positive, and 50 negative images were predicted negative using the sensitivity driven convolutional neural network model (Table 3). The overall accuracy of the sensitivity driven convolutional neural network model was 79.01% with a sensitivity of 73.68% and specificity of 80.64% (Table 6).

**TABLE 3:** Prediction result of the sensitivity driven model

	Predict positive	Predict negative	Total
True Positive	14	5	19
True Negative	12	50	62
Total	26	55	81

For board‐certified veterinary radiologists, of the 792 images in the entire data set, 208 positive images were predicted positive, 73 positive images were predicted negative, 64 negative images were predicted positive, and 447 negative images were predicted negative based on radiologists radiographic reports (Table 4).

**TABLE 4:** Performance of radiologists for the entire data set (n = 792)

	Radiologist positive	Radiologist negative	Total
True Positive	208	73	281
True Negative	64	447	511
Total	272	520	792

For board‐certified veterinary radiologists, of the 81 images in the testing dataset, the performance of radiologists was as follows: 13 positive images were predicted positive, 6 positive images were predicted negative, 8 negative images were predicted positive, and 54 negative images were predicted negative based on radiologists radiographic reports (Table 5). The accuracy of board‐certified radiologists interpretation of the testing dataset was 82.71% with a sensitivity of 68.42% and specificity of 87.09%. (Table 6).

**TABLE 5:** Performance of radiologists for the testing dataset (n = 81)

	Predict positive	Predict negative	Total
True Positive	13	6	19
True Negative	8	54	62
Total	21	60	81

**TABLE 6:** Performance comparison of accuracy driven model, sensitivity driven model, and board‐certified radiologist

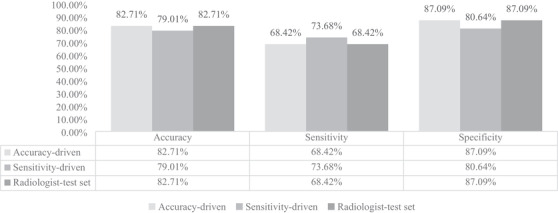

Table 6 shows the comparison of performance of the accuracy‐driven model, sensitivity‐driven model, and radiologists. The accuracy, sensitivity, and specificity from the accuracy driven model are identical to those from radiologists, however, the individual data points were interpreted differently.

The prediction result from accuracy driven convolutional neural network model was compared with the performance of radiologists. There was agreement of the convolutional neural network model prediction and radiologist determination in 69 of 81 cases resulting in 85% congruence. Among these 69 predictions, the convolutional neural network model and the radiologist were both incorrect in 8 instances and were both correct for the other 61 predictions. The false positive rate and false negative rate from convolutional neural network models and the radiologists were similar. Detailed prediction results are shown in Table 7.

**TABLE 7:** Congruency between accuracy driven convolutional neural network model and veterinary radiologists

	True positive	True negative	Agreement
Radiologist+/CNN+	12	3	Concordant
Radiologist‐/CNN‐	5	49	Concordant
Radiologist+/CNN‐	1	5	Discordant
Radiologist‐/CNN+	1	5	Discordant

Abbreviation: CNN, convolutional neural network.

**FIGURE 2:**
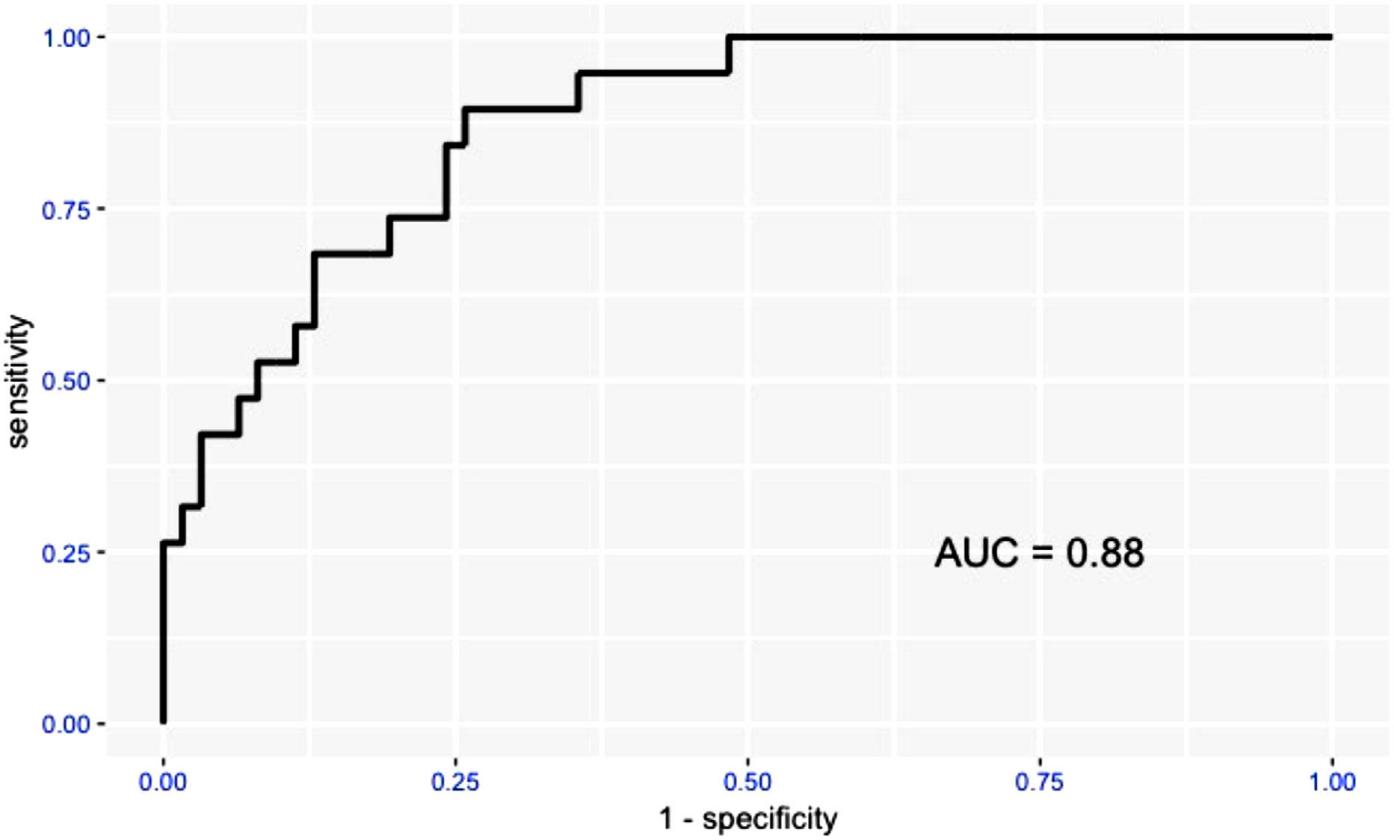
Receiver operating characteristic curve and areas under the curve of “Accuracy driven model” [Color figure can be viewed at wileyonlinelibrary.com]

**FIGURE 3:**
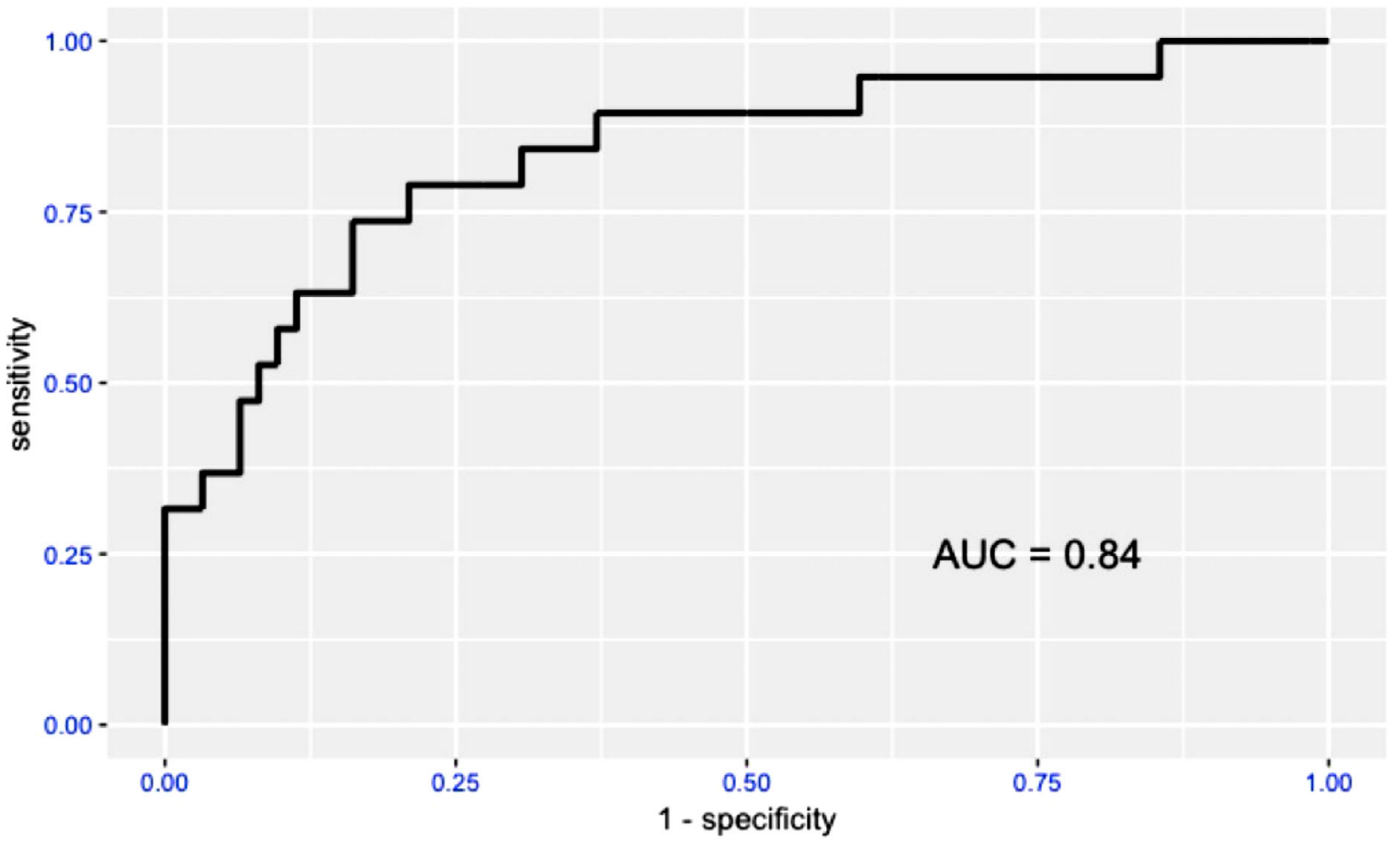
Receiver operating characteristic curve and areas under the curve of “Sensitivity driven model”

The receiver operating characteristic curves and areas under the curve for the accuracy driven convolutional neural network model and the sensitivity driven convolutional neural network model are shown in Figures 2 and 3. The area under the curve for the accuracy driven convolutional neural network model is 0.88; the area under the curve for the sensitivity driven convolutional neural network model is 0.84.

## 4 DISCUSSION

In this preliminary investigation, the convolutional neural network model was trained and validated using single right lateral radiographic images down‐sized to a 64 × 64 matrix size to keep the computational analysis to a reasonable scale given the available computing resources. Despite the fact that these data were compared to veterinary radiologist interpreted examinations that included three‐view, high resolution images and a pertinent patient history, the convolutional neural network model was able to consistently achieve similar accuracy and sometimes higher sensitivity in detection of left atrial enlargement. These findings are consistent with a recent meta‐analysis investigating performance of artificial intelligence versus clinicians in disease diagnosis.^21^

The application of artificial intelligence and computer‐aided diagnosis has recently gained considerable attention in human medicine with an emerging recognition that these technologies will dominate the field of medical imaging diagnosis in the not too distant future.^22^, ^23^, ^24^, ^25^, ^26^ By comparison, there is a paucity of veterinary literature and only a few studies were performed since the advent of robust convolutional neural network models currently available.^27^, ^28^, ^29^, ^30^, ^31^, ^32^ In the current investigation, detection of left atrial enlargement was used as a simple clinically relevant exemplar to illustrate the efficacy of deep learning to address common clinical imaging questions using a large archive of diagnostic images with known left atrial enlargement status based on echocardiographic results. As the database of canine thoracic radiographs and the deep learning‐based software platform are expanded and refined, the technique could also be applied to detection/diagnosis of pulmonary nodules, pneumonia, left ventricular failure, developmental cardiac anomalies, and other thoracic pathologic conditions in future investigations.^26^, ^33^, ^34^, ^35^, ^36^ By extension, creation of other databases using images from other imaging modalities and other anatomic regions could be used to address an endless number of clinical diagnostic questions.

The algorithm used for this study provides a percent likelihood of a positive result (presence of left atrial enlargement) for a given image. For convolutional neural network data of this type, there are a number of practical applications including flagging a patient's imaging study as “high‐risk” for follow‐up review by a specialist, serving as a second “over‐read” following a primary clinician or specialist interpretation or simply serving as a fully automated screening or diagnostic test. Although the latter alternative is not currently applicable, rapid advances in the field of artificial intelligence and computer‐aided diagnosis may make automated diagnosis feasible in the near future.^37^, ^38^, ^39^, ^40^

A number of clinically applicable approaches have been used to assess left atrial enlargement from thoracic radiographs including subjective evaluation of cardiac contours, tracheal bifurcation angle measurements, and vertebral left atrial size.^7^, ^8^ Yet none of these methods has been proven to be consistently accurate. This is, in large part, due to inherent limitations of the thoracic radiographic examination for assessment of left atrial enlargement. Confounding variations in the appearance of the cardiac silhouette are caused by breed variability, patient positioning differences, cardiac and respiratory phase, and cardiac and noncardiac co‐morbidities, among other parameters. Inconsistent assessment can also be the result of inter‐reader or intra‐reader interpretation variability. The use of echocardiographic findings as a standard for left atrial size has its own inherent limitation as the inaccuracy from echocardiograph will be transferred into the convolutional neural network model. One big potential problem of the convolutional neural network is the overfitting,^19^ in particular when the dataset is small. The dropout layer technique is applied to reduce it. The convolutional neural network model described in this study however provides an objective evaluation tool that can consistently improve itself by continually expanding the training dataset of echocardiograph‐validated images. The application of deep learning in detecting left atrial enlargement using this approach could potentially improve detection accuracy and possibly reduce “time to diagnosis” through automation.

As with any study using retrospectively derived data, there are a number of limitations to this investigation. Matrix down‐sizing, necessary to distill imaging data down to a manageable computational size, necessarily reduces quality of the data as does limiting analysis to a single right lateral view. We also recognize that changes in left atrial size could occur within 72 hours separating radiographic and echocardiographic examinations. Despite these limitations, as the amount of data used to train the model increases; including number of patients, the number of image views, and image matrix size; the convolutional neural network model might be expected to achieve higher accuracy, sensitivity, and specificity in the future.

## 5 CONCLUSION

Results from this preliminary investigation suggest that the convolutional neural network model is applicable to canine thoracic image analysis and computer‐aided diagnosis. The convolutional neural network model was shown to achieve accuracy and sensitivity similar to veterinary radiologists in detecting left atrial enlargement and has the potential to be modified to address other clinically pertinent diagnostic questions.

## LIST OF AUTHOR CONTRIBUTIONS

### Category 1

(a) Conception and Design: Li, Wisner, Cheng
(b) Acquisition of Data: Li, Visser, Wisner
(c) Analysis and Interpretation of Data: Li, Wang, Visser, Wisner, Cheng

### Category 2

(a) Drafting the Article: Li, Wang, Visser, Wisner, Cheng
(b) Revising Article for Intellectual Content: Li, Wang, Visser, Wisner, Cheng

### Category 3

(a) Final Approval of the Completed Article: Li, Wang, Visser, Wisner, Cheng

## CONFLICT OF INTEREST

The authors declare no conflict of interest.
